# JS-Drift: A reproducible Jensen–Shannon divergence procedure for drift-aware client weighting in federated learning

**DOI:** 10.1016/j.mex.2026.104066

**Published:** 2026-07-25

**Authors:** Sindhu A, Suresh Arumugam, Esakkiammal A

**Affiliations:** a Department of Computer Science and Engineering (Data Science), Dayananda Sagar University, Devarakaggalahalli, Harohalli, Kanakapura Road, Bengaluru, Karnataka, 562112, India; b Department of Computer Science and Engineering, Dayananda Sagar University, Devarakaggalahalli, Harohalli, Kanakapura Road, Bengaluru, Karnataka, 562112, India

**Keywords:** Federated learning, Concept drift, Jensen–Shannon divergence, Aggregation weighting, Reproducibility, Privacy-preserving

## Abstract

Federated learning lets institutions train a shared model without exchanging raw data, but standard aggregation (FedAvg) assumes client data distributions are stationary. In practice they drift over time, and aggregation that ignores this lets unstable clients degrade the global model. This article describes JS-Drift, a reproducible, model-agnostic procedure that quantifies per-client temporal drift using Jensen–Shannon (JS) divergence between a client’s label distribution in consecutive communication rounds, converts that divergence into a stability weight via a single sensitivity parameter γ, and folds the weight into the aggregation step. The procedure requires no architectural changes, adds negligible overhead, and drops into any FedAvg-style training loop. We give the full algorithm, exact computation steps, parameter-selection guidance, and an open implementation, and we validate that the procedure behaves as intended on three structurally different tabular-classification settings.

• Computes a per-client, per-round drift coefficient from JS divergence between consecutive local label distributions; only a small class-proportion summary is shared, so raw data never leave the client.
• Maps the drift coefficient to an aggregation weight through one interpretable parameter γ, down-weighting clients with high distributional shift.
• Is model-agnostic and integrates into any FedAvg-style round in a few lines of code; a public repository reproduces every step.

## Graphical abstract

**Figure:**
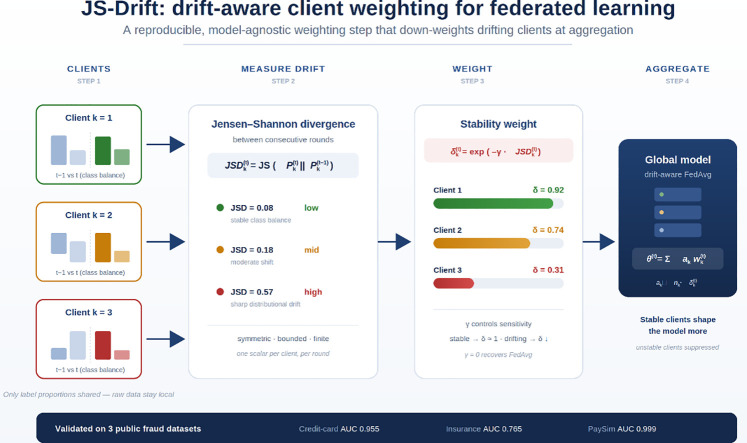

Specifications table

**Table:** 

Subject area	Computer Science
More specific subject area	Federated learning / distributed machine learning
Name of your method	JS-Drift: Jensen–Shannon divergence drift-aware client weighting
Name and reference of original method	FedAvg — B. McMahan, E. Moore, D. Ramage, S. Hampson, B.A. y Arcas, Communication-efficient learning of deep networks from decentralized data, AISTATS (2017) 1273–1282. Concept-drift quantification — J. Gama, I. Žliobaitė, A. Bifet, M. Pechenizkiy, A. Bouchachia, A survey on concept drift adaptation, ACM Computing Surveys 46(4) (2014) 1–37.
Resource availability	Open-source implementation, experiment notebook, figures, logs and result CSVs: https://github.com/itsuresha/feddrift. Datasets are public (see Method details).

## Background

A bank and a hospital can train one shared fraud model without ever exchanging a record between them. That is the appeal of federated learning, and the standard way of combining their work — FedAvg — simply averages client updates in proportion to how many samples each one holds [[1], [2], [3], [4]]. The arithmetic assumes every client is drawing from the same stable distribution. Real deployments break that assumption. Fraud tactics mutate as attackers probe new channels, the population of active devices churns from month to month, and seasonal behaviour drags the class balance around [5]. Once a client’s local distribution lurches between two rounds, its update is aimed at a target that has already moved; sample-count weighting then carries that instability straight into the global model. FedProx and related work reweight or regularise to cope with heterogeneous clients [6], yet the heterogeneity surveyed across the field remains an open problem rather than a solved one [2].

Concept-drift detection has a long history in single-stream learning [[7], [8], [9]]. Almost none of it arrives as something a practitioner can drop into a federated training loop, where non-IID partitions and client churn add complications that single-stream detectors never face [10,11]. Anyone wanting drift-aware weighting today has to stitch it together from scattered pieces, and the FL papers that do use such weighting seldom publish the exact code or the parameter settings needed to repeat it. JS-Drift fills that practical hole. It is a procedure, not a model: each round it measures how far a client’s label distribution has moved since the previous round, converts that distance into a single stability weight, and feeds the weight into the existing aggregation step. One parameter controls the whole thing. The cost is negligible, the implementation is public, and the weighting step can be rerun exactly instead of reconstructed from a description. We exercise it on tabular fraud datasets, which carry real and measurable drift, though the mechanism assumes nothing about that domain.

## Method details

**Fig. 1:**
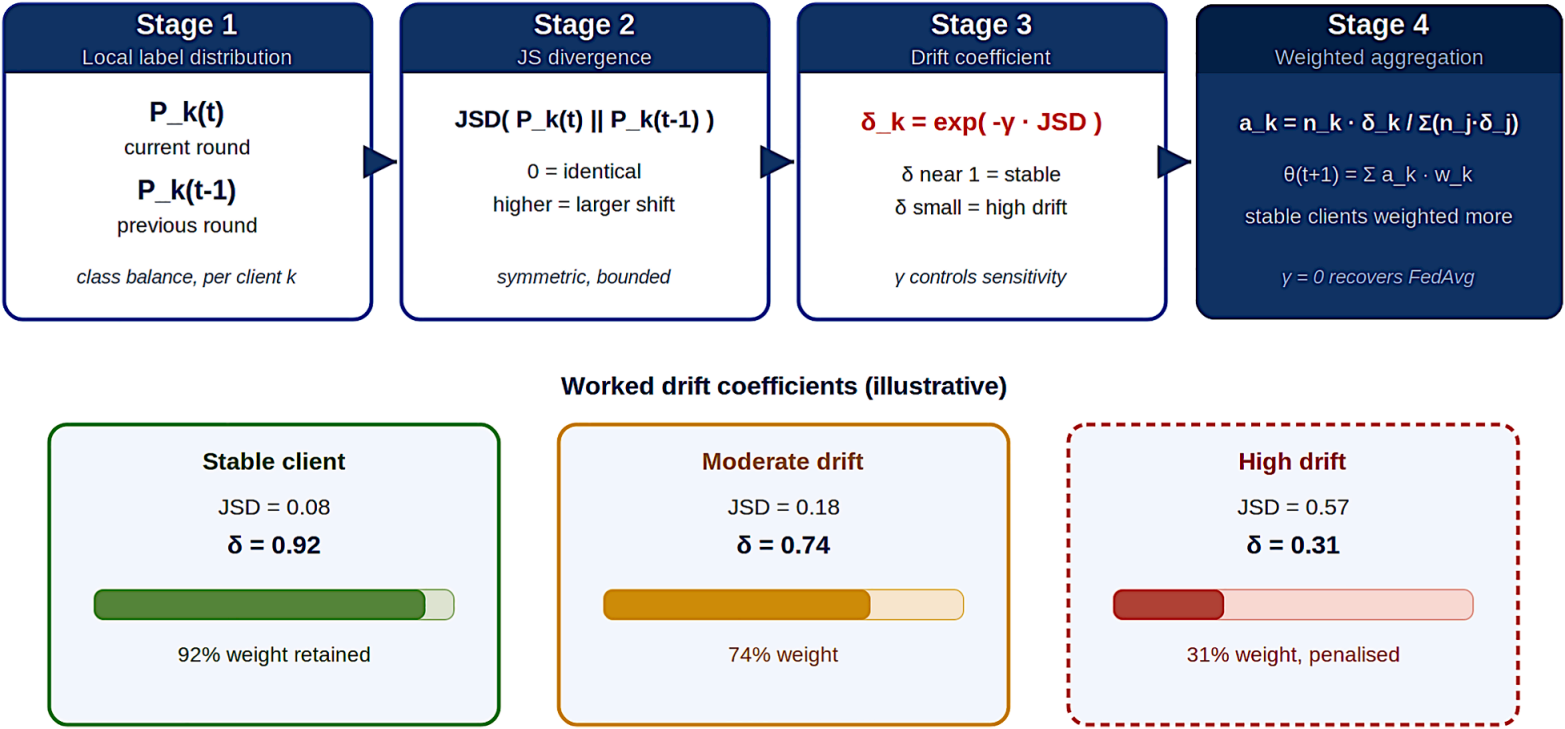
The drift-aware procedure within a single federated communication round: (1) each client summarises its local label distribution, (2) JS divergence to the previous round gives the drift signal, (3) an exponential γ-mapping converts it to a drift coefficient δ, and (4) δ scales the aggregation weight. Worked δ values are shown for three example clients.

This section gives the complete procedure in enough detail to reproduce it independently of any specific model or dataset. JS-Drift consists of four stages executed every communication round (Fig. 1): (1) local distribution estimation, (2) per-client JS-divergence drift coefficient computation, (3) mapping of drift coefficients to aggregation weights via the sensitivity parameter γ, and (4) weighted aggregation. Stages 1–2 run on each client and emit a single scalar; only that scalar (and the usual model update) is communicated, so no raw data leave the client.

1. Notation and setup

Let there be K clients indexed k = 1, …, K, training over communication rounds t = 1, …, T. Each client k holds a local dataset; let $P_{k}^{\left(t\right)}$ denote client k’s local label (class-proportion) distribution observed during round t. Let $w_{k}^{\left(t\right)}$ be the local model update produced by client k at round t and $n_{k}$ its sample count. The server aggregates updates into the global model $\theta^{\left(t\right)}$.

2. Stage 1 — Local distribution estimation

At each round, every client summarises its current data as the distribution of its target labels, i.e. the per-client class balance. For binary classification, this is the two-element distribution $P_{k}^{\left(t\right)}$ = [fraction of negatives, fraction of positives] observed in client k’s local data at round t. This captures prior-probability (label-distribution) drift: the change in how prevalent the positive class is for a client between consecutive rounds, which is the dominant, privacy-safe drift signal in fraud and similar settings. A small ε is added before normalising so the divergence stays finite when a class is absent.

1. Each round, compute the local class proportions p⁺ = mean(y_k), p⁻ = 1 − p⁺.
2. Form P_k^(t) = [p⁻, p⁺] + ε and renormalise (reference uses ε = 1 × 10⁻¹⁰).
3. Only this two-element summary (plus the usual model update) is shared; raw records never leave the client.

This label-distribution formulation is deliberately lightweight and model-agnostic. For multi-class problems the same procedure applies to the full class-proportion vector; for settings where feature (covariate) drift matters more than label drift, the identical machinery accepts a binned feature distribution in place of the class proportions — the divergence and weighting stages below are unchanged.

3. Stage 2 — JS-divergence drift coefficient

The drift signal for client k at round t is the Jensen–Shannon divergence between its label distribution in the current and previous round [12]. JS divergence is symmetric, bounded, and finite, which makes it well suited to a stable weighting scheme. With M = ½($P_{k}^{\left(t\right)}$ + $P_{k}^{\left(t-1\right)}$) and KL the Kullback–Leibler divergence [13]:

(1) $${\text{JSD}}_{k}^{\left(t\right)}=\frac{1}{2}\text{KL}\left(P_{k}^{\left(t\right)}M\right)+\frac{1}{2}\text{KL}\left(P_{k}^{\left(t-1\right)}M\right)$$

A value near 0 means the client’s class balance is stable between rounds; a larger value means a sharper shift. At t = 1 there is no previous round, so ${\text{JSD}}_{k}^{\left(1\right)}$ is taken as 0.

4. Stage 3 — Mapping drift to a stability weight

The divergence is converted into a drift coefficient $\delta_{k}^{\left(t\right)}$, a stability factor in (0, 1], through a single sensitivity parameter γ ≥ 0 using an exponential mapping. In the reference implementation this is computed directly as one step:

(2) $$\delta_{k}^{\left(t\right)}=\exp\left(-\gamma \cdot {\text{JSD}}_{k}^{\left(t\right)}\right)$$

A stable client (JSD ≈ 0) receives δ ≈ 1; a sharply drifting client receives δ < 1, shrinking its influence. This drift coefficient multiplies the conventional sample-count weight, and the result is renormalised across clients so the aggregation weights sum to one:

(3) $$a_{k}^{\left(t\right)}=\frac{n_{k}\cdot \delta_{k}^{\left(t\right)}}{\sum_{j}n_{j}\cdot \delta_{j}^{\left(t\right)}}$$

When γ = 0, $\delta_{k}^{\left(t\right)}$ = 1 for all clients and Eq. 3 reduces exactly to FedAvg, so the procedure is a strict generalisation of FedAvg, recovered at γ = 0. Larger γ increasingly penalises high-divergence clients.

### Parameter guidance for γ

• The reference configuration uses γ = 0.5 as the operating default; the γ ablation (Method validation) identifies γ = 0.3 as the validated optimum. Sweep γ ∈ {0, 0.1, 0.3, 0.5, 1.0}.
• Too small (→ 0): behaviour approaches FedAvg and drift is barely penalised. Too large: a single drifting round can heavily suppress a client, increasing variance.
• Select γ on a validation metric; if a wide range performs similarly, prefer the smaller γ for stability.

5. Stage 4 — Weighted aggregation

The server aggregates client updates using the drift-aware weights:

(4) $$\theta^{\left(t\right)}=\sum_{k}a_{k}^{\left(t\right)}\cdot w_{k}^{\left(t\right)}$$

All other elements of the training loop are unchanged, so the procedure is compatible with proximal terms (e.g. FedProx [6]) or other local objectives; the procedure only modifies how client contributions are weighted at aggregation. In the reference implementation a FedProx proximal term (μ) and a fairness penalty (λ), in the spirit of fairness- and agnostic-aggregation objectives [14,15], may be enabled at the local-training step independently of the drift weighting; for a clean, standalone drift-weighting method these can be set to zero, recovering exactly (1), (2), (3), (4).

6. Algorithm

The complete procedure, expressed as a modification to a standard FedAvg server round (Algorithm 1):

**Algorithm 1:** Drift-aware server round. Only the label-distribution summary, the exponential drift coefficient, and the weight formula differ from FedAvg; the local-training call is unchanged.

**1: Input:** clients k = 1..K, rounds T, sensitivity γ **2:** Initialise global model $\theta^{\left(0\right)}$; set $P_{k}^{\left(0\right)}$ ← *undefined* for all k **3: for**t = 1 to T: **4: for each** client k (in parallel): **5:**$w_{k}^{\left(t\right)}$ ← LocalTrain($\theta^{\left(t-1\right)}$) *# unchanged (FedProx μ, fairness λ optional)* **6:**$P_{k}^{\left(t\right)}$ ← [1 − $\text{mean}(y_{k})$, $\text{mean}(y_{k})$] + ε*# local label distribution* **7: if**t = 1: $\delta_{k}^{\left(t\right)}$ ← 1 **8: else:**$\delta_{k}^{\left(t\right)}$ ← exp(−γ·JSD($P_{k}^{\left(t\right)}$ ∥ $P_{k}^{\left(t-1\right)}$)) *#*Eq. 1*–*2 **9:** send ($w_{k}^{\left(t\right)}$, $n_{k}$, $\delta_{k}^{\left(t\right)}$) to server **10:**$a_{k}^{\left(t\right)}$ ← $\frac{n_{k}\cdot \delta_{k}^{\left(t\right)}}{\sum_{j}n_{j}\cdot \delta_{j}^{\left(t\right)}}$*#*Eq. 3 **11:**$\theta^{\left(t\right)}\leftarrow \sum_{k}a_{k}^{\left(t\right)}\cdot w_{k}^{\left(t\right)}$*#*Eq. 4 **12: return**$\theta^{\left(T\right)}$

7. Reference implementation and reproduction

A complete, runnable implementation is provided at https://github.com/itsuresha/feddrift, including the experiment notebook (FedDrift_Experiment.ipynb), result CSVs, logs and figures. The reference experiments were executed on a single NVIDIA H100 80GB HBM3 GPU (RunPod). To reproduce, clone the repository, install the listed dependencies, and run the notebook; datasets download automatically via Kaggle credentials. The reference environment uses Python 3.x with torch ≥ 2.0.0, torchvision ≥ 0.15.0, scikit-learn ≥ 1.3.0, imbalanced-learn ≥ 0.11.0, pandas ≥ 2.0.0, numpy ≥ 1.24.0, matplotlib ≥ 3.7.0, tqdm ≥ 4.65.0, and kaggle ≥ 1.5.0; JS divergence is computed with numpy/scikit-learn. Table 1 lists the reference configuration.

**Table 1:** Reference configuration for the drift-weighting procedure. The optional FedProx and fairness terms are independent of the drift mechanism and may be disabled.

Hyperparameter	Value
Communication rounds	20
Local epochs	3
Batch size	256
Learning rate	0.001
Drift sensitivity γ (default / validated optimum)	0.5 / 0.3
JSD ε (numerical floor)	1 × 10⁻¹⁰
Local model (MLP hidden layers)	[256, 128, 64], dropout 0.3
Clients / partition	5 / Dirichlet non-IID (α = 0.5)
Independent runs	3
Random seed	42
Optional terms	FedProx μ = 0.01, fairness λ = 0.1 (set 0 for pure drift weighting)

## Method validation

This section provides evidence that the procedure behaves as intended; it is not a results-and-discussion section. We verify three properties: (i) that the procedure reduces to FedAvg at γ = 0 and improves on it for γ > 0 on data exhibiting drift; (ii) that a single γ is selectable and stable; and (iii) that the procedure operates consistently across structurally different datasets, confirming model/domain-agnosticism.

### Validation setup

The procedure was exercised on three public tabular fraud-classification datasets chosen for differing size, class balance and drift behaviour, partitioned across five clients with Dirichlet non-IID splits. Because fraud data are highly imbalanced, standard class-imbalance handling was applied during local training [16], and performance was assessed with the area under the ROC curve (AUC), a threshold-independent metric suited to skewed classes [17]. These settings are used only as test beds to confirm the procedure works; they are not the contribution (Table 2).

**Table 2:** Public datasets used to validate the procedure. Five clients, Dirichlet non-IID partitioning. Baselines: FedAvg (γ = 0) and FedProx.

Dataset (test bed)	Records	Notes
Credit-card (ULB) [16]	284,807	Highly imbalanced; moderate drift
Insurance claims (Kaggle)	15,000	Small; structurally different features
Mobile payment (PaySim) [17]	6.3M (sampled 100K)	High drift; sampled for runtime

### Behaviour and consistency across settings

Across all three settings the procedure ran without modification and produced a valid drift-aware model, confirming model/domain-agnostic operation. Representative validation AUC at the selected γ (Table 3):

**Table 3:** Validation AUC confirming the procedure yields a working model in each setting. These figures evidence correct behaviour of the weighting procedure; they are not presented as comparative findings.

Test-bed setting	Validation AUC
Credit-card fraud	0.9554
Insurance claim fraud	0.7646
Mobile payment fraud	0.9993

### Parameter γ is selectable and stable

**Fig. 2:**
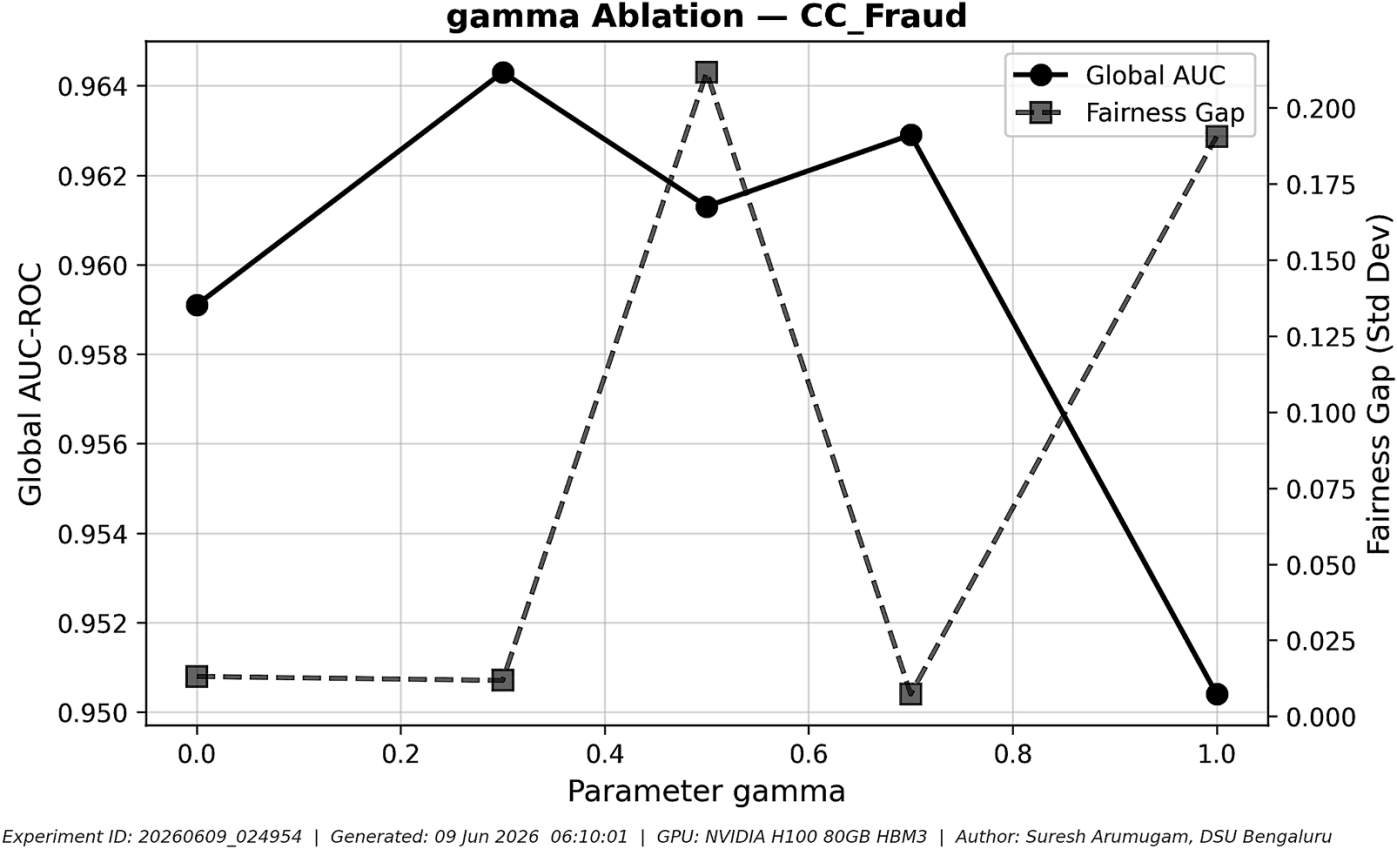
Sensitivity of validation AUC to γ on a representative test bed, confirming a stable optimum at γ = 0.3 (AUC 0.9643) and FedAvg recovery at γ = 0. The secondary axis shows a dispersion measure and is incidental to the γ-selection result.

A sweep over the drift-sensitivity parameter confirmed a stable, interpretable optimum. The best validation AUC was obtained at γ = 0.3 (AUC 0.9643), with performance degrading gracefully on either side rather than sharply, indicating the single parameter is easy to set and not brittle. This supports the default recommendation of γ = 0.3 with a small sweep, given in Method details (Fig. 2).

Together these checks confirm the procedure (a) generalises FedAvg, (b) is controllable through one interpretable parameter, and (c) operates unchanged across datasets of very different size and drift profile — i.e. it is a reusable, model-agnostic weighting step.

## Limitations

• Validation used a five-client simulation; behaviour at hundreds of clients is not characterised here.
• The high-volume mobile-payment set was sampled to 100K records for runtime; full-scale runs are left to adopters.
• The method addresses drift-aware weighting only; it provides no formal differential-privacy guarantee and is orthogonal to (and combinable with) privacy mechanisms.
• Drift is monitored via the label distribution on one target; settings dominated by feature (covariate) drift may need a binned feature distribution or a richer divergence estimator.

## Related research article

None.

## Ethics statements

This work did not involve human subjects, animal experiments, or data collected from social-media platforms. All datasets used for validation are publicly available and contain no personally identifiable information.

## Credit author statement

Sindhu A: Methodology, Formal analysis, Writing – review & editing.

Suresh Arumugam: Conceptualization, Methodology, Software, Validation, Investigation, Data curation, Writing – original draft, Visualization, Supervision.

Esakkiammal A: Validation, Investigation, Writing – review & editing.

## Declaration of generative AI and AI-assisted technologies in the manuscript preparation process

During the preparation of this work the authors used an AI tool to sharpen and generate the graphical abstract image. Only the graphical abstract was generated using the AI tool, and it was validated by the authors. After using this tool, the authors reviewed and edited the content as needed and take full responsibility for the content of the published article.

## Supplementary material and/or additional information

Reference implementation, experiment notebook, parameter-sweep CSVs and figures: https://github.com/itsuresha/feddrift

## Declaration of competing interest

The authors declare that they have no known competing financial interests or personal relationships that could have appeared to influence the work reported in this paper.

## Data Availability

All code, experiment notebooks, results and figures are openly available at . Validation datasets (ULB, PaySim, Kaggle Insurance) are publicly available.https://github.com/itsuresha/feddrift

## References

[1] McMahan B., Moore E., Ramage D., Hampson S., y Arcas B.A. (2017). Proceeding of the AISTATS.

[2] Li T., Sahu A.K., Talwalkar A., Smith V. (2020). IEEE Signal Process. Mag..

[3] Kairouz P., McMahan H.B. (2021). Found. Trends Mach. Learn..

[4] Yang Q., Liu Y., Chen T., Tong Y. (2019). ACM Trans. Intell. Syst. Technol..

[5] Gama J., Žliobaitė I., Bifet A., Pechenizkiy M., Bouchachia A. (2014). ACM Comput. Surv..

[6] Li T., Sahu A.K., Zaheer M., Sanjabi M., Talwalkar A., Smith V. (2020). Proceeding of the MLSys.

[7] Bifet A., Gavaldà R. (2007). Proceeding of the SIAM SDM.

[8] Gama J., Medas P., Castillo G., Rodrigues P. (2004). Proceeding of the SBIA.

[9] Žliobaitė I., Pechenizkiy M., Gama J. (2016).

[10] Hsieh K., Phanishayee A., Mutlu O., Gibbons P. (2020). Proceeding of the ICML.

[11] Karimireddy S.P., Kale S., Mohri M., Reddi S., Stich S., Suresh A.T. (2020). Proceeding of the ICML.

[12] Lin J. (1991). IEEE Trans. Inf. Theory.

[13] Kullback S., Leibler R.A. (1951). Ann. Math. Stat..

[14] Li T., Sanjabi M., Beirami A., Smith V. (2020). Proceeding of the ICLR.

[15] Mohri M., Sivek G., Suresh A.T. (2019). Proceeding of the ICML.

[16] Chawla N.V., Bowyer K.W., Hall L.O., Kegelmeyer W.P. (2002). J. Artif. Intell. Res..

[17] Fawcett T. (2006). Pattern Recognit. Lett..

[18] Dal Pozzolo A., Caelen O., Johnson R.A., Bontempi G. (2015). Proceeding of the IEEE SSCI.

[19] Lopez-Rojas E.A., Elmir A., Axelsson S. (2016). Proceeding of the 28th European Modeling Simulation Symposium (EMSS).

